# Delayed esophagopleural fistula after endoscopic injection sclerotherapy for esophageal varices: A case report

**DOI:** 10.1097/MD.0000000000018806

**Published:** 2020-01-17

**Authors:** Mingliang Sui, Weibing Tang, Changjiang Wu, Jinhu Yang, Huiping Liu, Chaofa Huang, Xianzhu Hu, Damei Xia, Yadi Yang

**Affiliations:** Department of Critical Care Medicine, Suzhou Kowloon Hospital, Shanghai Jiao tong University School of Medicine, Suzhou, China.

**Keywords:** case report, conservative treatment, endoscopic injection sclerotherapy, esophagopleural fistula

## Abstract

**Rationale:**

Esophagopleural fistula (EPF) is a rare critical life-threatening condition that features high misdiagnosis rate. Although various surgical and conservative techniques have been developed for the treatment of EPF, the mortality rate of EPF remains high.

**Patient concerns:**

An 81-year-old man with hepatic cirrhosis caused by schistosomiasis was admitted with upper gastrointestinal bleeding.

**Diagnoses:**

Upper endoscopy revealed bleeding large esophageal varices, and endoscopic injection sclerotherapy (EIS) was performed. Two weeks after the EIS was performed, the patient developed pyrexia, left-sided pleuritic chest pain. Air and pleural effusion were showed in the left pleural cavity by high-resolution computed tomography (HRCT), and a linear fistulous communication was noticed from the distal esophagus. These findings were consistent with hepatic cirrhosis, esophageal varices, upper gastrointestinal bleeding, and esophagopleural fistula.

**Interventions:**

The patient was intensively treated with endoscopic self-expandable metallic stent (covered-SEMS) implantation and comprehensive treatments (including thoracic closed drainage, antibiotics, nasojejunal nutrition, and antacids).

**Outcomes:**

The patient was completely cured without recurrence during a 6 months of follow-up by comprehensive conservative treatments.

**Lessons:**

This case indicates that pleural effusion with food residue is a specific finding in EPF. Thorax CT exhibited high sensitivity for the diagnosis of EPF. Endoscopic self-expandable metallic stent implantation and comprehensive conservative treatments may be preferable for the severe liver disease with EPF.

## 1 Introduction

Esophagopleural fistula (EPF) is a rare critical complication of endoscopic injection sclerotherapy (EIS).^[1]^ EPF can directly induce chemical inflammation and bacterial infection of the mediastinum, pleural cavity and lungs, and then causing acute mediastinal inflammation, severe pneumonia, or even sepsis, septic shock, and multiple organ dysfunction syndrome.^[2]^

Although various surgical and conservative techniques have been developed for the treatment of EPF, the mortality rate of EPF remains high (about 20%).^[3]^ Early diagnosis and timely treatment are crucial to improving the cure rate and reducing the mortality rate of EPF.^[4]^ The clinical manifestations of EPF are usually nonspecific, so EPF is more likely to be misdiagnosed as severe pneumonia, bacterial pleurisy, lung abscess, or thoracic stomach.^[5]^ A comprehensive understanding of the diagnosis and treatments for EPF is urgently needed.

## 2 Case presentation

**Figure 1:**
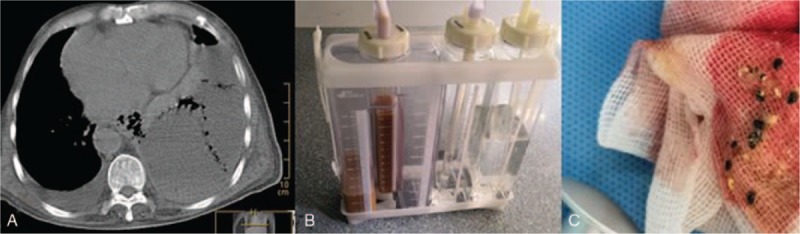
(A) HRCT image of the left pleural cavity showing pleural effusion with air in it. (B and C) Thoracic closed drainage confirmed food residue in it.

**Figure 2:**
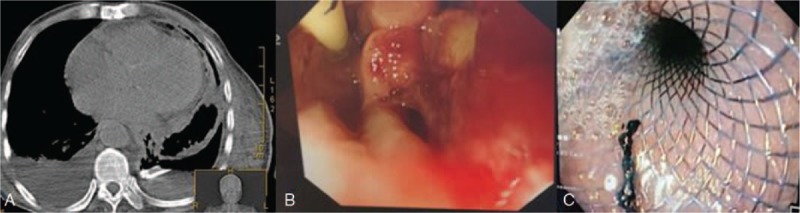
(A) The esophagopleural fistulous connection was more evident after thoracic closed drainage. (B) The upper gastrointestinal (GI) endoscopy confirmed a large fisulous tract measuring 6 mm at the distal esophagus. (C) Endoscopy images showing fully expanded stent.

**Figure 3:**
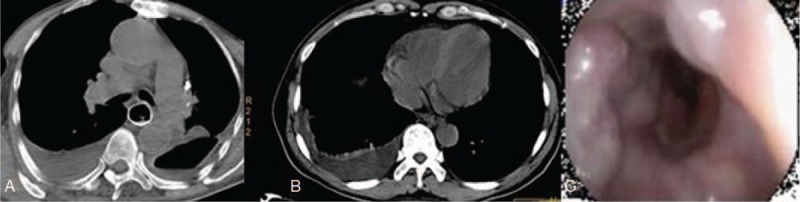
(A) CT image of the thorax showing a self-expandable metallic stent. (B) CT images showing complete closure of the esophagopleural fistula after stent removal. (C) Endoscopy images showing complete closure of the esophagopleural fistula 6 months later.

An 81-year-old man with hepatic cirrhosis caused by schistosomiasis was admitted with upper gastrointestinal bleeding. Upper endoscopy revealed bleeding large esophageal varices, and endoscopic injection sclerotherapy with 1% polidocanol 2.5 mL followed by tissue adhesive 0.5 mL was successfully performed by our endoscopist, and then the bleeding stopped. The procedure was uneventful. Two weeks after the EIS was performed, the patient developed pyrexia, left-sided pleuritic chest pain, and a chest radiograph demonstrated a left-sided pleural effusion. The chest pain was more severe than is usually seen after EIS. An encysted collection (9.2 cm × 12 cm × 13 cm) of air and fluid was noted in the left pleural cavity by high-resolution computed tomography (HRCT) with a linear fistulous communication from the distal esophagus (Fig. 1A). A chest tube was inserted for drainage, and gastric contents (food residue) were found in the pleural effusion (Fig. 1B and C). The esophagopleural fistulous connection was more evident after thoracic closed drainage (Fig. 2A). Upper endoscopy revealed a mucosal slough with a deep intramural tunnel (Fig. 2B) in the lower third of the esophagus, lined with granulation tissue. Pleural effusion cultures were positive for *Enterococcus faecium* and *Candida albicans*. Symptoms improved markedly within 2 weeks by an endoscopic self-expandable metallic stent (covered-SEMS) implantation (Figs. 2C and 3A) and comprehensive conservative treatments (including thoracic closed drainage, antibiotics, and nasojejunal nutrition). Two months after the stent insertion, the stent was removed by pulling removal snare with an alligator forceps. With a 6 months follow-up, the patient has recovered from fistula after the stent removal (Fig. 3B and C).

## 3 Discussion

Delayed esophagopleural fistula is a serious complication of EIS that features high mortality and high misdiagnosis rate in patients with severe liver disease.^[6]^ In adults, spontaneous esophageal rupture, pneumonectomy, esophageal foreign body, and iatrogenic trauma were revealed to be the main causes of EPF. In our case, the patient with EPF was revealed to be induced by EIS, which indicated that the iatrogenic damage may be an important etiology of EPF in patients with severe liver disease.

The early Symptoms of EPF were variable and atypical, and the misdiagnosis rate was usually high. To our knowledge, 36 cases (31 adults, 5 children) with EPF after EIS have been published to date. The time lag between the EIS and documentation of an EPF was 9 to 35 days.^[1–8]^ In literature, pleural effusion was the only manifestation of EPF (100%). Unilateral pleural effusion was more frequent than bilateral pleural effusion (53.0% right side, 39.5% left side, 7.5% bilateral). Meanwhile, 77.8% of cases of pleural effusion were accompanied with food residue. Fever (52.8%), dyspnea (58.3%), pectoralgia (44.34%), and subcutaneous and mediastinal emphysema (27.8%) were also found with EPF. Although various surgical and conservative techniques have been developed for the treatment of EPF, the mortality rate of EPF remains high, accounting for approximately 20%. Treatment and prognosis are largely determined by the time to presentation. Our patient presented 2 weeks after EIS, with similar symptoms including fever, severe thoracalgia, unilateral hydro-pneumothorax, and food residue in the pleural effusion. These phenomena indicate that greater attention should be paid to the properties of pleural effusion. Specifically, when gastric contents appear in pleural effusion, the diagnosis of EPF should be suspected clinically.

For an accurate diagnosis of EPF, chest radiograph, ultrasound, barium swallow, and HRCT are the most commonly used inspection methods. HRCT could reveal fistula size and location, which exhibited high diagnostic sensitivity on EPF (>90%).^[9]^ On thorax CT, the typical manifestation of EPF mainly included pleural and mediastinal effusion, esophageal fistula interlinked with the mediastinum, and pulmonary inflammation. Therefore, HRCT may be the diagonostic method of choice.

It is possible that sclerosants themselves may play a role in producing necrosis in the esophageal wall, because sodium morrhuate or ethanolamine oleate, a derivative of morrhuate, was the sclerosants used in the reports of esophageal complications.^[10]^ Injection of large volume of sclerosant could lead to an increased frequency of severe chest pain and an increased risk of transmural complications. In our case, the leak was located between the middle and lower thirds of the esophagus, possibly due to inadvertent injection higher in the esophagus. Symptoms starting 2 weeks after injection may support both an inflammatory and mechanical mechanism for perforation. Local friable mucosa after injection might also be a cause of such perforation. It is possible that severe liver disease with impairment in reparative ability may be another significant predisposing factor.

The choice of management of EIS-related EPF is controversial.^[11]^ Primary repair is often recommended when the perforation is diagnosed early; with a minor leak without communication to the pleural cavity, a conservative approach may be adequate. Endoscopic treatment (covered-SEMS) was suitable for delayed diagnosed EPF patients with poor health condition.^[12]^ In patients provided endoscopic covered-SEMS implantation, fully effective pleural, mediastinal drainage, anti-infective measures, and antacids were considered necessary to avoid digestive juice-induced chemical corrosion and thoracic mediastinal infection. Meanwhile, nasojejuna nutritional support should also be administered. In our case, surgical repair could not be performed for the advanced nature of the liver disease and poor condition. The patient was treated with endoscopic covered-SEMS implantation and comprehensive conservative treatments. This patient was completely cured without recurrence during a 6-month of follow-up by comprehensive conservative treatments. This case suggests that endoscopic covered-SEMS implantation and conservative treatment could be preferable for the severe liver disease cases with EPF.

## Author contributions

**Formal analysis:** Weibing Tang, Jinhu Yang.

**Project administration:** Mingliang Sui, Huiping Liu.

**Supervision:** Chaofa Huang, Xianzhu Hu.

**Writing – original draft:** Mingliang Sui, Changjiang Wu.

**Writing – review & editing:** Damei Xia, Yadi Yang.

Weibing Tang orcid: 0000-0002-5736-4257.
